# Membrane-free bone regeneration using biphasic calcium phosphate, heterologous fibrin biopolymer, and photobiomodulation: an *in vivo* study

**DOI:** 10.1590/1678-9199-JVATITD-2026-0012

**Published:** 2026-08-03

**Authors:** Bruna Trazzi Pagani, Filiphe Gustavo Daré, Beatriz de Oliveira Fernandes, Karina Oliveira Santos, Benedito Barraviera, Rui Seabra Ferreira, Murilo Priori Alcalde, Marco Antonio Hungaro Duarte, Daniela Vieira Buchaim, Rogerio Leone Buchaim

**Affiliations:** 1 School of Dentistry, University of Marilia (Unimar), Marília, SP, Brazil.; 2 Department of Biological Sciences, Bauru School of Dentistry (FOB), University of São Paulo (USP), Bauru, SP, Brazil.; 3 Center for the Study of Venoms and Venomous Animals (Cevap), São Paulo State University (Unesp), Botucatu, SP, Brazil.; 4 Professional Graduate Program in Clinical Research, Botucatu Medical School (FMB), São Paulo State University (Unesp), Botucatu, SP, Brazil.; 5 Center for Translational Science & Biopharmaceuticals Development (CTS), Center of the Study of Venoms and Venomous Animals (Cevap), São Paulo State University (Unesp), Botucatu, SP, Brazil.; 6 Department of Dentistry, Endodontics and Dental Materials, Bauru School of Dentistry (FOB), University of São Paulo (USP), Bauru, SP, Brazil.; 7 Medical School, University Center of Adamantina (FAI), Adamantina, SP, Brazil.; 8 Graduate Program in Anatomy of Domestic and Wild Animals, Faculty of Veterinary Medicine and Animal Science (FMVZ), University of São Paulo (USP), São Paulo, SP, Brazil.; 9 Graduate Department, School of Dentistry, Faculty of the Midwest Paulista (Facop), Piratininga, SP, Brazil.

**Keywords:** Biocompatible materials, Bone regeneration, Photobiomodulation, Fibrin sealant, Biopolymers

## Abstract

**Background:**

Although bone tissue possesses inherent regenerative capacity, critical-sized defects require grafts for complete functional repair. This *in vivo* study evaluated the bone repair process using laser photobiomodulation therapy (PBM) in defects filled with a combination of hydroxyapatite, β-tricalcium phosphate and heterologous fibrin biopolymer (HFB).

**Methods:**

Thirty male rats were divided into three groups: biomaterial alone (BG), biomaterial + HFB (BBG), and biomaterial + HFB + PBM (BBPG). A 5-mm circular calvarial osteotomy was performed and filled according to each protocol. In BBPG, an 830-nm laser was applied immediately post-surgery and three times weekly until euthanasia at 14 or 42 days. Analyses included micro-CT, histomorphology, histomorphometry, and polarized light microscopy of collagen fibers.

**Results:**

Micro-CT showed centripetal bone regeneration restricted to defect margins, with biomaterial particles persisting centrally. Histologically, new bone progressed from immature trabecular architecture at day 14 to a mature lamellar conformation by day 42, notably in BBPG. All groups showed a significant temporal increase in new bone percentage. BBPG demonstrated superior bone growth at 42 days (26.64 ± 2.15%) compared to BG (14.85 ± 1.63%) and BBG (20.05 ± 1.70%). The birefringence of the collagen fibers showed a color transition from red to yellowish-green during the analyzed periods.

**Conclusion:**

The combination of the biomaterial, fibrin biopolymer and photobiomodulation significantly enhanced bone defect repair and matrix maturation without barrier membranes, presenting high translational potential for cost-effective clinical applications in regenerative medicine.

## Background

When bone tissue sustains an injury, it possesses an inherent capacity to regenerate, ultimately restoring both its architecture and functionality. The healing process begins immediately with the inflammatory phase, marked by clot formation [1, 2]. During this stage, various signaling molecules, including inflammatory mediators, angiogenic agents, and growth factors, are released, activating immune cells [3]. As collagen networks develop, they provide a scaffold for the formation of new bone, initially producing what is known as primary or immature bone [4]. This is followed by the remodeling phase, in which bone resorption and regeneration gradually reshape the tissue until its original structure and function are fully reestablished [5].

Bone defects differ in terms of shape, size, mechanical integrity, and vascularization, factors that can either facilitate or impede the healing process [6]. Minor defects typically heal without complication, whereas critical-size defects (CSDs) often lead to the development of fibrous tissue rather than new bone, compromising full regeneration [7]. Bone restoration may be necessary in a range of clinical contexts, including traumatic injuries, congenital abnormalities, and the surgical removal of tumors [8, 9].

In cases involving extensive bone defects, the application of implants and bone substitutes becomes essential to support the healing process [10, 11]. Grafting techniques are employed to enhance or accelerate local regeneration, using bone sourced from various origins: autografts (from the patient), allografts (from a donor of the same species), or xenografts (from a different species) [12]. Although autogenous grafts are considered the gold standard due to their biocompatibility and osteogenic potential, their use is constrained by the need for dual surgical procedures and associated risks [13, 14]. As a result, research has focused on developing synthetic alternatives, including alloplastic biomaterials such as hydroxyapatite, beta-tricalcium phosphate (β-TCP), bioactive polymers, and specialized bioglasses designed to mimic the properties of natural bone [15-19].

To promote satisfactory repair, certain bioproducts or biopharmaceuticals are required to provide a three-dimensional scaffolding system for the biomaterials, such as fibrin biopolymer (also called heterologous fibrin sealant). This material is composed of thrombin and fibrin, which, in addition to providing this biological scaffold, also acts as a sealant, hemostatic, and healing agent in the biological processes of tissue repair [20-23].

To accelerate morphofunctional recovery, physical therapies such as low-level laser therapy (LLLT), now more commonly referred to as photobiomodulation therapy (PBM), play a significant role in bone repair [24]. PBM utilizes low-intensity light, typically within specific wavelength ranges, to target injured tissues, exerting biomodulatory effects at the cellular level [25, 26]. In the context of bone defect healing, PBM has been shown to reduce inflammation, promote vascular proliferation, and stimulate osteoblast activity and osteogenesis [27, 28]. These effects collectively contribute to faster and higher-quality regeneration of the treated area [29-31].

Previous studies integrating bone repair therapies with heterologous fibrin and photobiomodulation have been carried out with various objectives, such as the stabilization of autogenous bone grafts [32], the evaluation of new biomaterials in guided bone regeneration (GBR) using membranes as epithelial growth barriers, in rat calvaria [33, 34] or long bone repair under different photobiomodulation protocols [35, 36]. In the present study, a photobiomodulation protocol was combined with a particulate synthetic biomaterial and a 100% heterologous fibrin biopolymer purified from snake venom, without the use of protective barriers such as the membranes of GBR membranes.

This experimental protocol was designed to evaluate the bone regeneration process using photobiomodulation (PBM) therapy by integrating and analyzing the key components involved in effective tissue repair. The study focused on defects treated with a composite material combining hydroxyapatite and β-tricalcium phosphate, which was further enhanced by the addition of a heterologous fibrin biopolymer to form a novel biocomplex.

## Methods

### Experimental design

Thirty adult male Wistar rats (*Rattus norvegicus*), aged 90 days and with a mean body weight of approximately 285 g, were obtained from the Central Animal Facility of the University of Marília (Unimar). The experimental protocol was reviewed and approved by the Institutional Animal Care and Use Committee (CEUA-Unimar), under protocol number 007/2018.

Upon arrival, the animals were housed in standard polypropylene cages, with four rats per cage, under controlled environmental conditions. The bioterium maintained a 12-hour light/dark cycle regulated by an automated timer, with artificial illumination. Ambient temperature was stabilized at 22 ± 1 °C using air conditioning and an exhaust ventilation system, and monitored daily using a calibrated room thermometer. This study was conducted in accordance with the ARRIVE guidelines (Animal Research: Reporting of *In Vivo* Experiments), ensuring transparency and reproducibility in animal research. All procedures adhered to the ethical principles established by the National Centre for the Replacement, Refinement, and Reduction of Animals in Research (NC3Rs), aiming to minimize animal use and suffering while maximizing scientific integrity.

All animals were randomly assigned to three experimental groups to ensure unbiased distribution:

- biomaterial (BG);
- biomaterial + fibrin biopolymer (BBG);
- biomaterial + fibrin biopolymer + PBM (BBPG)

### Surgical Procedures

For the experimental surgical intervention, animals were anesthetized via intramuscular administration of a combination of tiletamine hydrochloride and zolazepam hydrochloride (10 mg/kg; Telazol^®^, Fort Dodge Laboratories, IA, USA). All procedures were conducted under the supervision of a licensed veterinary professional to ensure compliance with ethical and welfare standards.

Prior to surgery, the cranial region, specifically the frontal-parietal area between the external auricular pinnae, was shaved using an electric clipper (Philips^®^ Multigroom QG3250, São Paulo, Brazil). Body weight was recorded using a precision scale (MicroNal^®^, São Paulo, Brazil). Individual identification was performed by perforating the external ear pinnae with Ainsworth^®^ punch pliers (Golgran^®^, São Paulo, Brazil). The shaved region, including adjacent fur, was disinfected using a 10% topical solution of polyvinylpyrrolidone-iodine (PVPI; Povidine^®^, Vic Pharma Indústria e Comércio Ltda, São Paulo, Brazil).

Each surgical procedure was carried out independently on a sterile, cork-covered wooden surface, with all instruments replaced between specimens to prevent cross-contamination.

Animals were gently restrained in the prone position on the surgical table, ensuring minimal stress or trauma. A 4-cm semilunar skin incision was made using a No. 15 carbon steel scalpel blade (Embramax^®^, São Paulo, Brazil). The periosteum was carefully elevated with a syndesmotome and retracted along with surrounding soft tissues to expose the parietal bone surface.

A standardized circular osteotomy (5.0 mm diameter) was performed at the center of the parietal bone using a trephine drill (Neodent^®^, Curitiba, Brazil) mounted on a 500 Kavo electric contra-angle handpiece (KaVo^®^ Dental Excellence, Joinville, Brazil), connected to an electric micromotor (KaVo^®^ Dental Excellence, Joinville, Brazil). The procedure was executed at low rotational speed (1500 rpm) under continuous irrigation with sterile 0.9% saline solution to mitigate thermal damage and prevent bone necrosis. The resulting bone fragment was smooth-edged and intact, with preservation of the dura mater and underlying cerebral structures.

In the BG group, cranial defects were filled with a blood clot combined with the GenPhos XP^®^ (Baumer, Mogi Mirim, Brazil) biomaterial. In contrast, animals in the BBG and BBPG groups received a composite of heterologous fibrin biopolymer and GenPhos XP^®^ biomaterial. The biomaterial was weighed using an analytical precision balance (MicroNal^®^ Equipamentos de Precisão, São Paulo, Brazil) to ensure a standardized mass of approximately 0.04 mg. Following complete polymerization of the fibrin biopolymer with the biomaterial, the resulting biocomplex was carefully transferred to the defect site without exerting pressure on the underlying brain tissue.

Surgical site closure involved meticulous repositioning of the soft tissues, ensuring that the periosteum adequately covered the defect area. The skin was sutured using simple interrupted stitches with 4-0 silk suture material (Ethicon^®^, Johnson & Johnson, São Paulo, Brazil). The region was then gently cleansed with gauze moistened in a 2% topical chlorhexidine solution (Riohex^®^, Farmacêutica Rioquímica, São José do Rio Preto, Brazil).

Postoperatively, animals were placed in lateral recumbency within individual cages and exposed to incandescent light to facilitate anesthetic recovery. Immediately following surgery, a single dose of antibiotic (Flotril^®^ 2.5%, Schering-Plough, Rio de Janeiro, Brazil) was administered intramuscularly at 0.2 mL/kg. Analgesia was initiated with intramuscular dipyrone (Analgex V^®^, Agener União, São Paulo, Brazil) at a dose of 0.06 mL/kg and maintained for three consecutive days. Subsequently, analgesic support was continued with oral paracetamol (Medley^®^, São Paulo, Brazil) at a dose of 200 mg/kg, administered as six drops per animal diluted in drinking water until the time of euthanasia.

Throughout the experimental period, animals were closely monitored for signs of pain and distress. Behavioral assessments included evaluation of apathy, depression, aggression, or hyperexcitability, particularly when these traits deviated from baseline behavior. Activity levels were observed for changes ranging from hypoactivity to hyperactivity, with attention to gait, posture, and facial expression. Additional parameters included general appearance, food and water intake, clinical symptoms, spontaneous behavior, and responses to external stimuli.

### GenPhos XP^®^ biomaterial

GenPhos XP^®^ (Baumer S.A., Mogi Mirim, Brazil) represents a fully synthetic biphasic calcium phosphate ceramic, formulated as 70% hydroxyapatite [HA; Ca₁₀(PO₄)₆(OH)₂] and 30% β-tricalcium phosphate [β-TCP; Ca₃(PO₄)₂] granules (0.50-0.75 mm), characterized by interconnected macroporosity (70-240 µm) and a tailored resorption profile of 7-9 months for guided bone regeneration in dentoalveolar and maxillofacial procedures.

The biphasic matrix couples the inherent structural resilience and osteoconductive mimicry of the bone's mineral component, ensuring scaffold longevity, with the accelerated hydrolytic breakdown of β-TCP's under osteoclast- and macrophage-derived acidity, thereby promoting neovascularization and sequential substitution by autologous bone matrix [31].

### Heterologous fibrin biopolymer (HFB)

The heterologous fibrin biopolymer was manufactured and provided by the Center for the Study of Venoms and Venomous Animals at Unesp (Cevap, Botucatu, Brazil). Its components and application formulas are listed in patent BR 102014011432-7, issued on July 6, 2022, by the Brazilian National Institute of Industrial Property. The biopolymer consists of three separate solutions, previously thawed, mixed, and homogenized before application. Fraction 1 is thrombin-like (gyroxin), obtained from the venom of *Crotalus durissus terrificus*; the diluent is calcium chloride; and fraction 2 is fibrinogen (cryoprecipitate) from the blood of *Bubalus bubalis* (buffalo). The proportion used was 1:1:2 (fraction 1, diluent and fraction 2, respectively).

For each animal, the preparation of the heterologous fibrin biopolymer followed a standardized protocol. Initially, the GenPhos XP^®^ biomaterial was weighed using an analytical balance and stored in an Eppendorf Tubex^®^ 3810x microtube (Eppendorf AG, Hamburg, Germany). Subsequently, 10 µL of fraction 1 (thrombin-like enzyme) was pipetted into a separate microtube. In the second microtube, 10 µL of the calcium chloride diluent was combined with 20 µL of fraction 2 (fibrinogen derived from *Bubalus bubalis*), and the mixture was gently homogenized.

The contents of both microtubes were then transferred to a sterile plastic container to initiate polymerization. To prevent cross-contamination and ensure precision, the micropipette tip was replaced for each individual solution during the preparation process.

### Photobiomodulation (PBM)

Animals assigned to the BBPG group received photobiomodulation therapy using a gallium-aluminum-arsenide (GaAlAs) laser device. To facilitate precise irradiation of the calvarial region, animals were gently restrained manually, allowing full exposure of the surgical site without the need for anesthetic intervention.

The laser protocol employed continuous wave emission at a wavelength of 830 nm, with an output power of 30 mW and an energy density of 6 J/cm². Irradiation was performed at four equidistant points arranged in a cross-shaped configuration over the defect area, with each site receiving 24 s of exposure. The laser beam had a spot size of 0.116 cm², resulting in a power density of 258.6 mW/cm². Each irradiation point received 0.72 J of energy (24 s exposure), totaling 2.88 J per session across four points. The emitter was positioned in direct contact with the skin at a perpendicular angle (90°), yielding a total application time of 96 s per session.

Treatment commenced immediately after surgery and was administered three times per week until the endpoint of the experiment. Laser output calibration was performed directly on the device (Laserpulse^®^, IBRAMED, Amparo, Brazil) prior to each session. The therapeutic parameters were based on the protocol established by de Oliveira Gonçalves et al. [32], and utilized in previous research [33, 34], ensuring methodological consistency with prior studies.

### Sample collection and euthanasia

At 14 and 42 days postoperatively, five animals from each experimental group were weighed and subsequently euthanized. The procedure was conducted in a calm and isolated environment to minimize stress and avoid visual or auditory exposure to other animals. Euthanasia was performed via intraperitoneal administration of sodium thiopental (2.5% solution) at a dose of 150 mg/kg, applied to the lower left abdominal quadrant. To enhance analgesic coverage, lidocaine hydrochloride was co-administered at a dose of 10 mg/kg.

Following euthanasia, the calvarial defect region was carefully excised using a conical carbide burr mounted on a low-speed surgical handpiece (Dabi Atlante^®^, Ribeirão Preto, Brazil), ensuring preservation of the supraperiosteal soft tissues. The harvested specimens were immediately immersed in 10% neutral buffered formalin (pH 7.2) for fixation over a period of seven days. After fixation, samples were forwarded for histological processing.

Confirmation of death was followed by proper disposal procedures: the carcasses were placed in white biological waste bags, frozen, and sent to an authorized disposal facility in accordance with institutional biosafety protocols.

### Microtomography

Microtomographic images of each specimen were acquired using a SkyScan 1174 computed microtomography system (Bruker-microCT^®^, Kontich, Belgium). The reconstruction process was performed with the 64Bits270013 platform and the NRecon^®^ software (version 1.6.8.0, SkyScan, 2011, Bruker-micro-CT^®^), generating approximately 1000-1100 axial slices per sample, based on standardized anatomical reference parameters [35].

### Histomorphology and histomorphometry 

Following specimen collection, samples were rinsed under running water for 24 h and subsequently subjected to a demineralization protocol using an EDTA-based solution. The solution was composed of 4.13% Tritiplex^®^ III (Merck KGaA, Hessen, Germany) and 0.44% sodium hydroxide (Labsynth, São Paulo, Brazil), with weekly replacement over a six-week period. To monitor the progression of demineralization, radiographic assessments were performed during each solution change using Insight IP-21 F-Speed periapical film (Carestream^®^, Carestream Health, New York, USA).

Upon completion of demineralization, specimens were dehydrated through a graded ethanol series, cleared in xylene, and embedded in Histosec^®^ paraffin (Merck, Hessen, Germany). Semi-serial coronal sections were obtained from the central region of the defect using a Leica^®^ RM2245 semi-automatic microtome (Leica Biosystems^®^, Wetzlar, Germany). Sections were cut at a thickness of 5 µm, with six slides prepared per specimen, each containing four sections. These were stained with hematoxylin and eosin (HE), Masson’s trichrome (MT) and Picrosirius red (PR) for histological evaluation.

Histomorphological analysis was performed across all specimens, encompassing the entire defect area to assess bone regeneration patterns. Parameters evaluated included the presence of granulation tissue, inflammatory infiltrate, and the quality and maturity of newly formed bone, as well as the extent of defect filling by regenerated tissue.

For quantitative assessment, four semi-serial sections from each surgical site were examined using an Olympus^®^ light microscope (Olympus Corporation, Tokyo, Japan). Images were captured using a 10× objective lens coupled to a digital camera, with acquisition software configured to a resolution of 4080 × 3072 pixels and a spot resolution of 30%. Image analysis was conducted using ImageJ^®^ software version 1.50d (Wayne Rasband^®^, National Institutes of Health, USA; Java 1.7_67, 64-bit).

From the semi-serial sections, two central slices, representing the largest diameter of the defect and spaced 300 µm apart, were selected for morphometric analysis. The area of newly formed bone was measured, and the percentage of bone regeneration was calculated [36].

### Statistical analysis

Statistical analyses were conducted using GraphPad Prism version 8 (GraphPad Software Inc., 2018; San Diego, CA, USA). To evaluate the temporal effect on the percentage of newly formed bone within each experimental group, an unpaired Student’s t-test was applied. Intergroup comparisons at identical time points were performed using one-way analysis of variance (ANOVA) for independent samples, followed by Tukey’s post hoc test to identify specific differences. Statistical significance was established at a threshold of *p* < 0.05 for all tests. Each group and time point comprised five samples (n = 5). Prior to hypothesis testing, Bartlett’s test was employed to assess the homogeneity of variances and confirm the normal distribution of the data.

## Results

### Microtomography

**Figure 1.**
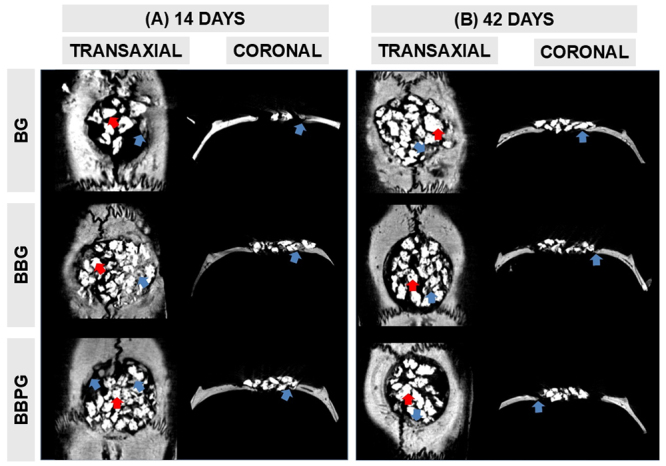
Representative micro-computed tomographic (micro-CT) images displaying transaxial and coronal views of calvarial bone defects across experimental groups at **(A)** 14 and **(B)** 42 days post-surgery. BG: defects treated with biomaterial alone; BBG: defects treated with biomaterial combined with heterologous fibrin biopolymer; BBPG: defects treated with biomaterial and heterologous fibrin biopolymer, followed by photobiomodulation therapy. Blue arrows indicate newly formed bone tissue, while red arrows denote biomaterial particles within the defect site. 5-mm osteotomy.

At the 14-day mark, two-dimensional micro-computed tomography (micro-CT) scans in both transaxial and coronal planes revealed findings consistent with histological analysis. All samples exhibited a centripetal pattern of bone regeneration, characterized by increased radiodensity at the lateral margins of the defect. Hyperdense biomaterial particles were visible in grayscale, occupying the surgical site (Figure 1 A).

By 42 days, all experimental groups showed deposition of partially mineralized bone tissue adjacent to the dura mater. However, this bone formation remained localized to the defect borders throughout the observation period. Biomaterial particles persisted in all specimens, and complete osseous closure of the defect was not achieved (Figure 1 B ).

### Histomorphology

**Figure 2.**
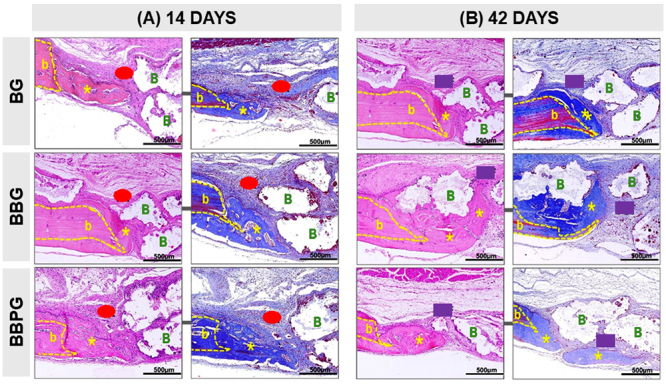
Histological sections of calvarial bone defects stained with hematoxylin and eosin (HE) and Masson’s trichrome (MT) at **(A)** 14 and **(B)** 42 days post-surgery. Across all experimental groups, newly formed bone tissue (indicated by asterisks) was consistently observed at the defect margins (yellow b). Reactive tissue (red ellipse) was present surrounding biomaterial particles (green B) at both time points. By day 42, the defect areas were predominantly occupied by fibrous connective tissue (purple rectangle), residual biomaterial, and a sparse distribution of inflammatory cells. Images captured at 10× magnification.

At 14 days post-intervention, histological analysis across all experimental groups revealed a consistent pattern of bone regeneration, initiating from the defect margins and progressing centripetally toward the wound center. The newly formed bone exhibited a trabecular architecture, densely populated by osteocytes and lined with active osteoblasts. The central region of the defect was predominantly occupied by granulation tissue, characterized by a high cellular content including inflammatory cells, neovascular structures, fibroblasts, and dispersed biomaterial particles (Figure 2 A).

By day 42, all specimens demonstrated complete coverage of the defect by fibrous connective tissue. Biomaterial particles were either encapsulated by dense collagen fibers or integrated into the mineralized bone matrix. The inflammatory infiltrate appeared sparsely distributed, indicative of a resolving inflammatory response. In the BBPG animals, the newly formed bone displayed a lamellar structure, suggestive of advanced maturation and ongoing remodeling. The surrounding connective tissue exhibited a pronounced collagenous composition (Figure 2 B ).

**Figure 3.**
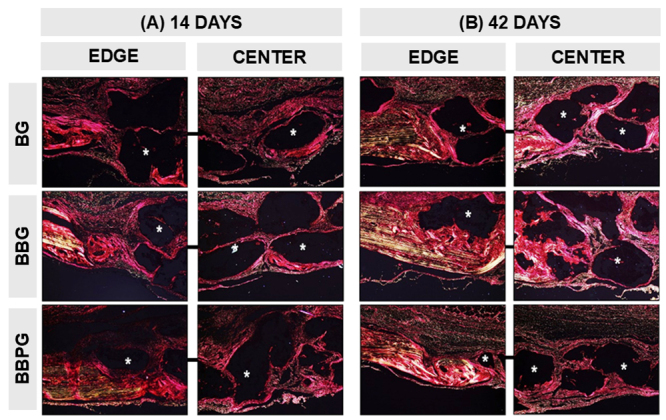
Polarized light microscopy images illustrate the birefringence of collagen fibers in coronal sections of rat calvarial bone defects, captured at the defect margins and central regions. Samples were stained with Picrosirius red and analyzed at **(A)** 14 and **(B)** 42 days post-surgery. BG: defects treated with biomaterial alone; BBG: defects treated with biomaterial combined with heterologous fibrin biopolymer; BBPG: defects treated with biomaterial and heterologous fibrin biopolymer, followed by photobiomodulation therapy. Biomaterial particles are indicated by asterisks. Images acquired using a 10× objective on an inverted light microscope.

Picrosirius red (PR) staining, examined under polarized light microscopy, enabled assessment of the maturation dynamics of the newly formed bone matrix. At 14 days, all specimens exhibited thin type I collagen fibers within the nascent bone matrix, displaying intense red birefringence. Notably, in the photobiomodulation group (BBPG), the collagen fibers appeared more organized and thicker, with a yellowish-red hue, indicative of advanced matrix maturation (Figure 3 A ).

By 42 days, the initial type I collagen fibers laid down during the proliferative phase were progressively replaced by broader type III fibrils, as evidenced by a shift toward yellow-green birefringence under polarized light. Residual GenPhos XP^®^ biomaterial particles observed at the conclusion of the experimental period appeared as darkfield structures (Figure 3 B ).

### Histomorphometric

**Figure 4.**
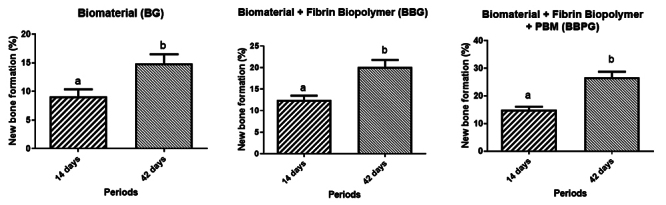
Quantitative analysis of new bone formation (%) in each experimental group (BG, BBG, and BBPG) at 14 and 42 days post-surgery. Data are presented as mean ± standard deviation. Distinct lowercase letters denote statistically significant differences between groups and time points (*p* < 0.05), as determined by an unpaired Student's *t*-test.

A statistically significant increase in the percentage of new bone formation was observed across all experimental groups (BG, BBG, and BBPG) when comparing the two time points - 14 and 42 days post-intervention (Figure 4).

**Figure 5.**
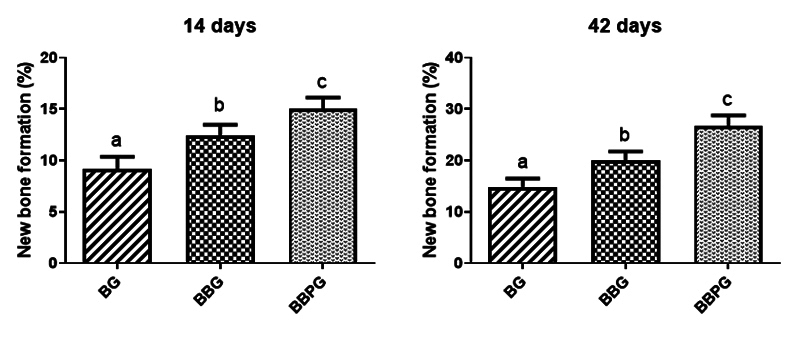
Percentage of new bone formation in each experimental group (BG, BBG, and BBPG) at 14 and 42 days post-surgery. Data are expressed as mean ± standard deviation. Distinct lowercase letters indicate statistically significant differences (*p* < 0.05), as determined by one-way ANOVA for independent samples followed by Tukey’s post hoc test.

Comparative analysis of the percentage of newly formed bone revealed statistically significant differences among all experimental groups (BG, BBG, and BBPG) at both 14 and 42 days (Figure 5 and Table 1; *p* < 0.05).

**Table 1.** Percentage of new bone formation in each group (BG, BBG and BBPG) in across the two experimental periods (14 and 42 days).

Groups	14 days	42 days	**Between-group *p* values at 14 days**	**Between-group *p* values at 42 days**
**BG**	9.12 ± 1.24	14.85 ± 1.63	vs. BBG: *p* = 0.0001; vs. BBPG: *p* < 0.0001	vs. BBG: *p* = 0.0001; vs. BBPG: *p* < 0.0001
**BBG**	12.45 ± 1.03	20.05 ± 1.70	vs. BG: *p* = 0.0001; vs. BBPG: *p* < 0.05	vs. BG: *p* = 0.0001; vs. BBPG: *p* = 0.0001
**BBPG**	15.02 ± 1.10	26.64 ± 2.15	vs. BG: *p* < 0.0001; vs. BBG: *p* < 0.05	vs. BG: *p* < 0.0001; vs. BBG: *p* = 0.0001

Data are expressed as mean ± standard deviation. One-way ANOVA indicated significant differences among groups at 14 days (F = 34.32, *p* = 0.0001) and 42 days (F = 51.24, *p* = 0.0001). Pairwise comparisons among groups at each time point were performed using Tukey’s multiple comparisons test.

## Discussion

This study evaluated *in vivo* the repair of a critical-sized calvarial bone defect using a heterologous fibrin biopolymer as a three-dimensional scaffold combined with a high-purity, synthetic biphasic calcium phosphate ceramic graft (70% hydroxyapatite and 30% β-tricalcium phosphate) manufactured in Brazil, without the use of traditional guided bone regeneration (GBR) barrier membranes. Additionally, this study investigated the local therapeutic effects of adjunctive photobiomodulation (PBM) therapy to accelerate the healing cascade.

Micro-computed tomography (micro-CT) was utilized to qualitatively monitor the progression of bone regeneration within the critical-sized defects. At 14 days post-surgery, an increased radiodensity was evident along the lateral margins of the defects, accompanied by hyperdense biomaterial particles occupying the surgical site. These particulate scaffolds facilitated centripetal bone formation, which occurred in close structural association with their surfaces. By day 42, the deposition of partially mineralized bone matrix was noted at the defect edges, aligning with findings from previous investigations utilizing structurally similar biphasic ceramics [37-39]. Overall, complete osseous closure was not achieved in any specimen by the 42-day mark. This incomplete closure is an expected biological outcome given the critical nature of the 5.0-mm defect model, which inherently lacks the capacity for spontaneous physiological healing within the animal's lifespan [40, 41].

Histological evaluation remains a foundational method for assessing the qualitative characteristics of tissue healing and new bone architecture. In the present study, histological analysis at 14 days revealed abundant granulation tissue characterized by inflammatory cell infiltration, active neovascularization, fibroblast proliferation, and dispersed biomaterial particles occupying the central region of the defect. By day 42, the inflammatory response had subsided across all groups, indicating a physiological progression toward a resolving remodeling phase. Notably, specimens from the BBPG group exhibited a more densely collagenized connective tissue matrix and newly formed bone with lamellar structural features, reflecting an advanced stage of bone maturation. These observations corroborate established literature indicating that the seamless structural integration of newly formed bone with residual biomaterial particles serves as an index of active osteoregenerative activity [42-44]. Furthermore, these qualitative histological findings strongly support the microtomographic and quantitative histomorphometric analyses.

The progression of bone neoformation was quantitatively assessed through histomorphometric analysis. A statistically significant temporal increase in the percentage of newly formed bone was observed across all experimental groups between the 14- and 42-day time points. Notably, the tri-therapeutic approach in the BBPG group demonstrated superior bone regeneration, yielding mean values of 15.02 ± 1.10% at day 14 and 26.64 ± 2.15% at day 42. These findings underscore the enhanced therapeutic efficacy achieved by combining advanced bioproducts (biphasic ceramic and fibrin scaffold) with PBM therapy, reinforcing the potential of this combinatorial protocol to accelerate bone matrix deposition within challenging clinical defect models [22, 45, 46].

The extracellular matrix plays a pivotal role in regulating cellular signaling, recruitment, and behavior during bone healing, with its structural integrity largely dependent on the organization of its collagenous components. In this study, the maturation dynamics of the newly formed bone matrix were assessed using polarized light birefringence imaging of Picrosirius red-stained coronal sections. At the 14-day time point, the collagen matrix exhibited predominantly thin type I collagen fibers, characteristic of the proliferative phase of inflammation [47, 48]. In contrast, specimens from the photobiomodulation group (BBPG) displayed highly organized, thicker collagen bundles, indicating accelerated matrix remodeling [26, 49]. By day 42, a gradual transition was observed across the groups, with type I fibers being increasingly replaced by broader type III fibrils [50]. These patterns demonstrate that adjunctive physical therapies, such as PBM, facilitate rapid collagen cross-linking and enhance the structural organization of the regenerating osteoid matrix [51, 52].

The BBPG group received photobiomodulation therapy utilizing a gallium-aluminum-arsenide (GaAlAs) laser. This modality is widely recognized in the literature for its ability to enhance angiogenesis and stimulate osteoblast proliferation, thereby promoting osteogenesis [53]. Additionally, it exerts anti-inflammatory effects at the local site, collectively contributing to accelerated and more effective bone regeneration within the biostimulated region [54].

One potential limitation of this study is the absence of a negative control group left to heal with a blood clot alone. However, it is well established that critical-size defects are fundamentally incapable of spontaneous regeneration, even when surgically stabilized, making a therapeutic intervention mandatory to evaluate active regeneration [55]. Furthermore, animal allocation into the experimental groups was guided by the ethical principles of the "3Rs" (replacement, reduction, and refinement) to minimize animal numbers while ensuring statistical validity [56]. These principles continue to serve as a cornerstone in contemporary biomedical research, ensuring responsible and humane use of animal models.

While a previous study by our research group confirmed the separate utilities of these modalities [33], the structural and biological interaction of this particulate biomaterial with both the heterologous fibrin biopolymer and PBM, specifically in the absolute absence of traditional guided bone regeneration barriers, remained to be elucidated. Eliminating costly collagen or synthetic membranes [57] could substantially reduce the financial burden of maxillofacial and dental bone grafting procedures, making advanced bone regeneration therapies more accessible.

## Conclusions

This study demonstrated the novel combinatorial application of a heterologous fibrin biopolymer (HFB), hydroxyapatite/β-tricalcium phosphate, and laser photobiomodulation (PBM) therapy for bone repair in the absolute absence of traditional guided bone regeneration (GBR) barrier membranes. 

Based on the qualitative micro-computed tomography, structural histomorphology, quantitative histomorphometry, and polarized light birefringence imaging of collagen fibers, it can be concluded that the combination of biphasic calcium phosphate with heterologous fibrin biopolymer and laser photobiomodulation therapy exerts a beneficial effect on bone regeneration. The combined protocol significantly accelerated bone matrix deposition and structural maturation within critical-sized calvarial defects. These findings highlight its high translational potential as a promising, cost-effective, and efficient therapeutic strategy in regenerative medicine and bone grafting procedures.

## Data Availability

All data generated or analyzed during this study are included in this article.
